# Cuticular protein with a low complexity sequence becomes cross-linked during insect cuticle sclerotization and is required for the adult molt

**DOI:** 10.1038/srep10484

**Published:** 2015-05-21

**Authors:** Seulgi Mun, Mi Young Noh, Neal T. Dittmer, Subbaratnam Muthukrishnan, Karl J. Kramer, Michael R. Kanost, Yasuyuki Arakane

**Affiliations:** 1 Department of Applied Biology, Chonnam National University, Gwangju, Korea; 2 Department of Biochemistry and Molecular Biophysics, Kansas State University, Manhattan, Kansas, United States of America

## Abstract

In the insect cuticle, structural proteins (CPs) and the polysaccharide chitin are the major components. It has been hypothesized that CPs are cross-linked to other CPs and possibly to chitin by quinones or quinone methides produced by the laccase2-mediated oxidation of *N*-acylcatechols. In this study we investigated functions of TcCP30, the third most abundant CP in protein extracts of elytra (wing covers) from *Tribolium castaneum* adults. The mature TcCP30 protein has a low complexity and highly polar amino acid sequence. TcCP30 is localized with chitin in horizontal laminae and vertically oriented columnar structures in rigid cuticles, but not in soft and membranous cuticles. Immunoblot analysis revealed that TcCP30 undergoes laccase2-mediated cross-linking during cuticle maturation *in vivo*, a process confirmed *in vitro* using recombinant rTcCP30. We identified TcCPR27 and TcCPR18, the two most abundant proteins in the elytra, as putative cross-linking partners of TcCP30. RNAi for the *TcCP30* gene had no effect on larval and pupal growth and development. However, during adult eclosion, ~70% of the adults were unable to shed their exuvium and died. These results support the hypothesis that TcCP30 plays an integral role as a cross-linked structural protein in the formation of lightweight rigid cuticle of the beetle.

The insect cuticle or exoskeleton is a complex extracellular biocomposite secreted by the epidermis. It consists of multiple functionally and morphologically distinct layers including the outermost waterproofing envelope, the protein-rich epicuticle and the innermost chitin-rich procuticle^1^,^2^. Two different structural biopolymers, cuticular proteins (CPs) and chitin, are the major components of the exo- and endocuticular layers of the procuticle. An insect must periodically replace its old cuticle with a new one by undergoing ecdysis (molting), because the cuticle is too restrictive to allow for continuous growth. Immediately after molting, the cuticle is soft and pale, but shortly thereafter it becomes hardened and often darker. During cuticle tanning (sclerotization and pigmentation), some of the CPs are cross-linked by quinones or quinone methides produced by the oxidation of *N*-acylcatechols catalyzed by laccase 2^3^,^4^,^5^. This vital process occurs during each stage of development. Expression of specific CPs is probably required for formation of diverse cuticles in different regions of the insect body and at different developmental stages, with appropriate combinations of physical and morphological properties to provide structural support, mechanical protection, and mobility. However, there is only limited information about the functional importance of individual insect CPs on morphogenesis and mechanical properties of the cuticle.

Bioinformatics analyses of fully sequenced and annotated genomes of several insect species have revealed the occurrence of a large number of genes encoding CP-like proteins in insect genomes^6^,^7^,^8^,^9^,^10^,^11^,^12^,^13^. Many of the insect CPs are classified into distinct families as defined by the presence of specific amino acid sequence motifs^12^,^14^. The CPR family is the largest, which includes CPs that have a conserved amino acid sequence known as the Rebers & Riddiford (R&R) motif^15^ that can function as a chitin-binding domain and help to coordinate the interactions between chitin fibers and the protein matrix^16^,^17^. The CP proteins belonging to the CPR family are further divided into three groups denoted as RR-1, RR-2 and RR-3 based on variations in their consensus amino acid sequences of their R&R motifs^7^,^12^,^18^,^19^. CPR proteins containing the RR-1 motif have been found mainly in relatively soft and flexible cuticles, while proteins containing the RR-2 motif have been found mostly in hard and rigid cuticles^11^,^20^. A small number of genes encoding CPR proteins with the RR-3 motif have been identified in only a few insect species^8^,^9^,^19^.

We previously identified two abundant CPs in adult *T. castaneum*, denoted as TcCPR27 and TcCPR18, in protein extracts of elytra (modified, hardened forewings that cover the folded hindwings)^21^. These two proteins are members of the RR-2 group of the CPR family. TcCPR27 and TcCPR18 proteins are abundant not only in dorsal elytral cuticle but also in other body regions with rigid cuticle such as the pronotum, ventral abdomen and leg, whereas they are absent (or present only in very minor amounts) in soft and flexible cuticles such as the ventral elytron, hindwing and dorsal abdomen of adult *T. castaneum*^21^. Furthermore, TEM immunogold labeling revealed that TcCPR27 protein is localized in both chitinous horizontal laminae and vertical pore canals in the procuticle of rigid adult cuticle^22^. dsRNA-mediated down-regulation of transcripts (RNAi) for *TcCPR27* and *TcCPR18* genes caused malformed elytra. In particular, the elytra of TcCPR27-deficient adults were shorter, wrinkled, fenestrated and less rigid than those of control insects, resulting in dehydration-induced mortality approximately one week after adult eclosion.

In this study, we focused on the third most abundant structural cuticular protein, TcCP30, present in protein extracts of elytra from adult *T. castaneum*. *TcCP30* cDNA encodes a protein with low sequence complexity and a unique amino acid composition, which lacks an R&R consensus motif. We examined the expression pattern, localization and function of TcCP30 in the production of rigid cuticle and in molting.

## Results and Discussion

### Identification of an abundant elytral cuticle protein

**Figure 1:**
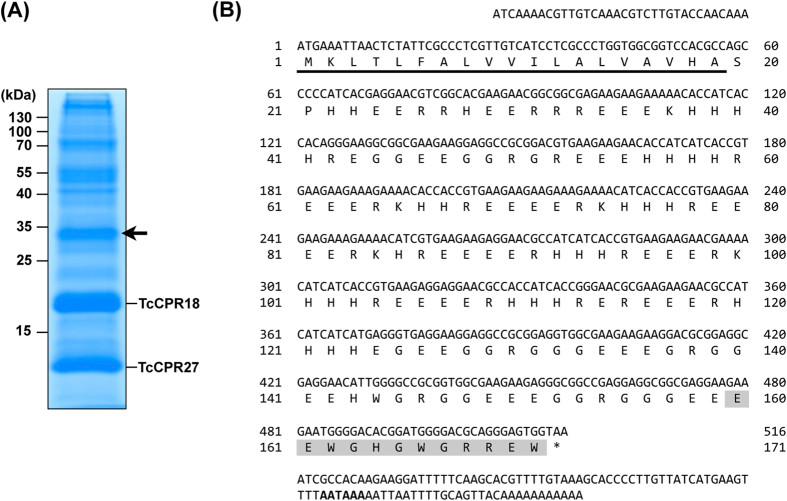
Identification of a major extractable non-RR cuticular protein TcCP30 in elytra. (**A**) Proteins extracted from elytra dissected from pharate adults (day 5 pupae) were analyzed by 15% SDS-PAGE. Arrow indicates TcCP30, the third most abundant protein, with an apparent molecular mass of 30 kDa. The other two abundant RR-2 cuticular proteins, TcCPR27 and TcCPR18, are also indicated^21^. (**B**) Nucleotide sequence of a full-length cDNA clone and the deduced amino acid sequence of TcCP30 encoded by this cDNA. The underline indicates predicted signal peptide. The peptide sequence used for TcCP30 antibody generation is highlighted in gray. The putative polyadenylation signal (AATAAA) is indicated in bold.

As reported previously^21^, SDS-PAGE analysis of protein extracts of elytra dissected from pharate adults (day 5 pupae) of *T. castaneum* exhibited two major proteins, TcCPR27 and TcCPR18 (Fig. 1A). These proteins contain an RR-2 motif^14^,^15^, and are present not only in dorsal elytral cuticle but also in thoracic and ventral abdominal cuticles that become highly sclerotized and pigmented in the mature adult. In this study, we focused on the third most abundant protein in the extracts, which had been identified by MALDI-TOF mass spectrometry of the peptides produced by trypsin treatment (protein spot E43 in 2D PAGE from Fig. 1 in Dittmer *et al.*^11^). We cloned a full-length cDNA for this cuticular protein and denoted it as TcCP30. *TcCP30* cDNA encodes a protein with 171 amino acid residues including an N-terminal putative secretion signal peptide (Fig. 1B). The mature TcCP30 protein lacks an R&R consensus motif. Instead, it has a low complexity amino acid sequence with a very unusual amino acid composition of 36% Glu, 21% His, 19% Arg and 16% Gly. The mature TcCP30 has a theoretical molecular mass of 19.0 kDa and pI of pH 5.8. Its electrophoretic mobility in SDS-PAGE is variable depending on the gel composition and electrophoresis buffers used. In 15% acrylamide gels for SDS-PAGE in Tris-glycine buffer (25 mM Tris, 192 mM glycine, 0.1% SDS), it migrates with an apparent size of 30 kDa (arrow in Fig. 1A). However, when using NuPAGE 4-12% gradient gels and MES buffer, the apparent mass of TcCP30 and recombinant rTcCP30 is ~25 kDa (see Supplementary Fig. 3A). TcCP30 has alternating blocks of 3-5 positively or negatively charged amino acid residues in the central portion of the protein, which might cause it to migrate abnormally during SDS-PAGE, and may also promote self-assembly in the cuticle through electrostatic interactions. This amino acid arrangement is unique among cuticular proteins described to date but a similar partial sequence was found in the giant northern termite, *Mastotermes darwiniensis* (GenBank accession number GAZE01460444.1). This finding suggests that homologous proteins may be present in other insect species (See Supplementary Fig. S1). TcCP30 has no predicted secondary structure, suggesting that it may be intrinsically disordered, and there are no predicted glycosylation sites. The *TcCP30* gene gives rise to a transcript derived from a single exon and is located on linkage group 5 of the *T. castaneum* genome.

### Expression of TcCP30 during development

The developmental pattern of expression of *TcCP30* transcripts was determined by quantitative RT-PCR. *TcCP30* transcripts were undetectable at the embryonic, larval, pharate pupal and mature adult stages of development (Supplementary Fig. 2A). The transcripts of the *TcCP30* gene, however, dramatically increased in pharate adults at five days after pupation, the day of adult eclosion under our insect rearing conditions, and it declined shortly thereafter eclosion (Supplementary Fig. 2B). The transcript abundances of the *TcCPR27* and *TcCPR18* genes were highest in day 4 pupae^21^, one day earlier than the peak of *TcCP30* mRNA expression. Like the *TcCPR27* and *TcCPR18* genes, the transcript level of *TcCP30* in the elytra was significantly higher (>4500-fold) than that in the membranous hindwings (Supplementary Fig. 2C)^21^. This difference is consistent with the result from microarray analysis of *T. castaneum* elytra and hindwings reported by Dittmer *et al.*^11^.

### RNA interference (RNAi) of *TcCP30*

**Figure 2:**
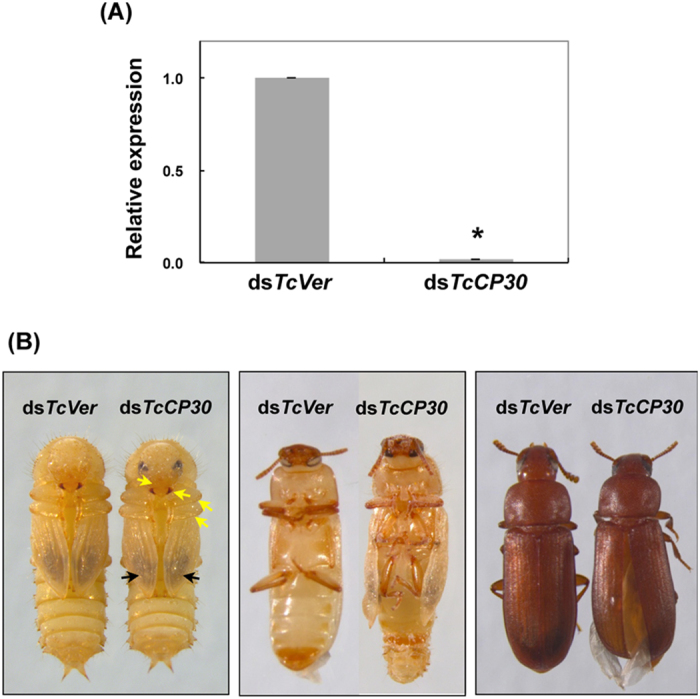
Loss of function phenotypes produced by RNAi for *TcCP30*. dsRNA for *TcCP30* or *TcVer* (200 ng per insect) was injected into late stage larvae. (**A**) The knockdown level of *TcCP30* transcripts was analyzed by real-time PCR using cDNAs prepared from total RNA isolated from pools of three day 5 pupae. Expression levels of *TcCP30* were presented relative to the level in ds*TcVer*-injected control insects. The transcript levels of *T. castaneum* ribosomal protein S6 (*TcRpS6*) were measured to normalize for differences in the concentration of cDNA templates between samples. An asterisk indicates a significant difference in transcript levels of *TcCP30* between control and test insects (p = 1.6E-05, t-test). Data are shown as mean value ± SE (n = 3). (**B**) Left panel shows absence of effect of RNAi for *TcCP30* on adult cuticle pigmentation including that of adult body wall, head, mandibles and legs (yellow arrows) as well as the hindwing including the pterostigma (black arrows), as observed through the pupal cuticle at the pharate adult stage (day 5 pupae). However, ~70% of the adults could not shed their old pupal cuticle and died without undergoing full eclosion (middle panel). Adults that did eclose exhibited split elytra (right panel).

**Figure 3:**
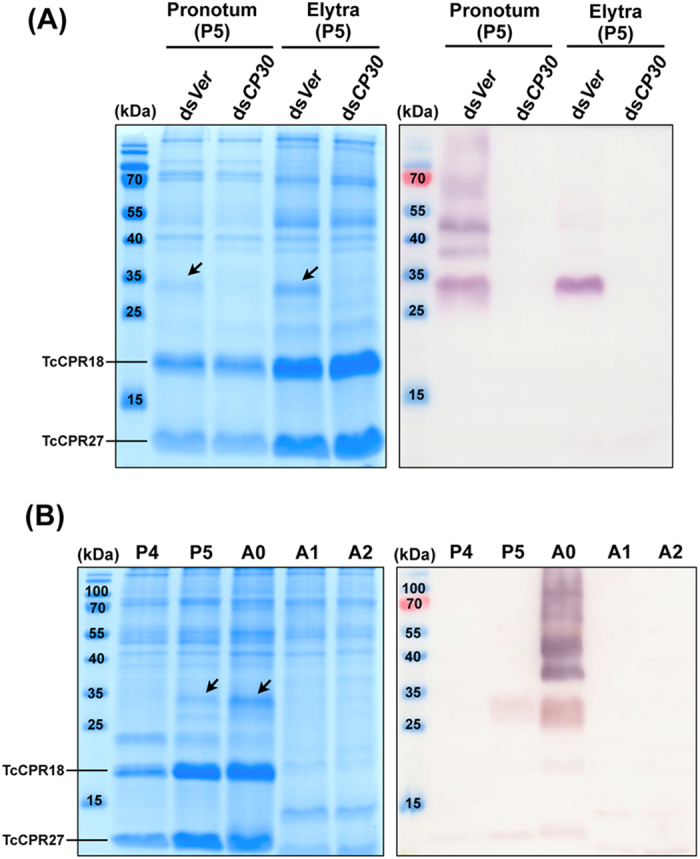
Specificity of anti-TcCP30 antibody and evidence for cross-linking of TcCP30. (**A**) Proteins were extracted from pronotum and elytra dissected from five ds*TcCP30*- (ds*CP30*) and from ds*TcVer* (ds*Ver*)-treated pharate adults (day 5 pupae, P5). (**B**) Proteins were extracted from elytra of late developmental stages (day 4 pupae to day 2 adults) of wild-type insects (n = 5). Equivalent amounts of the extracts were analyzed by 15% SDS-PAGE, followed by Coomassie staining (left panels in A and B) or by western blotting (right panels in A and B), respectively. Arrows indicate TcCP30 protein. P4, day 4 pupae; P5, day 5 pupae; A0, day 0 adults; A1, day 1 adults; A2, day 2 adults.

**Figure 4:**
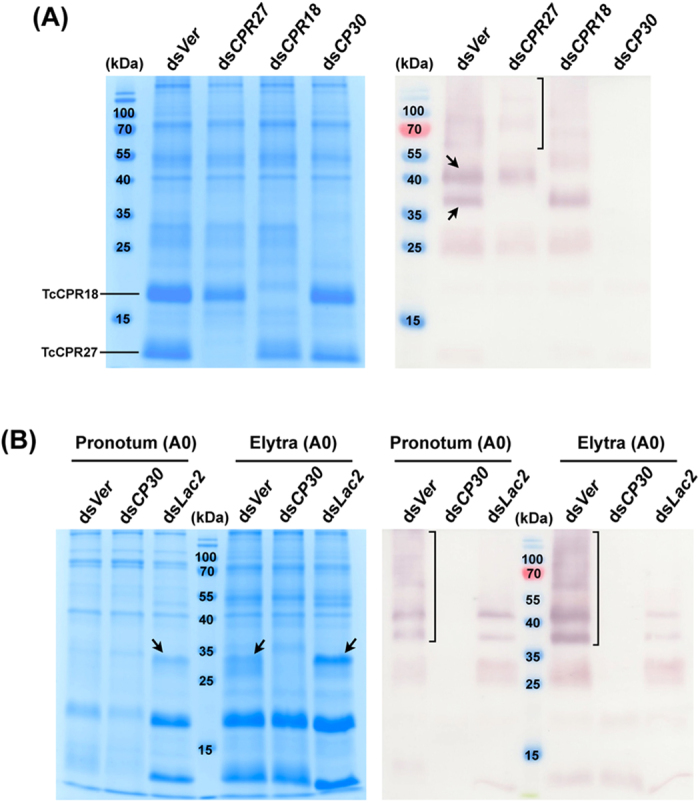
TcCP30 is involved in cross-linking. (**A**) TcCP30 cross-linking with TcCPR27 and TcCPR18. Proteins extracted from elytra dissected from five ds*TcCPR27* (ds*CPR27*)-, ds*TcCPR18* (ds*CPR18*)-, ds*TcCP30* (ds*CP30*)- or ds*TcCPR4* (ds*CPR4*)-treated insects (200 ng per insect) were collected at the day 0 adult stage and analyzed by 15% SDS-PAGE, followed by Coomassie staining (left panel) or western blotting (right panel). Arrows in right panel indicate large TcCP30 immunoreactive proteins with apparent molecular masses of ~37 and ~45 kDa. dsRNA for *TcVer* (ds*Ver*) (200 ng per insect) was injected as a negative control. (**B**) Lac2 is involved in TcCP30 cross-linking. dsRNAs for *TcCP30* (ds*CP30*) (200 ng per insect) or *TcLac2* (ds*Lac2*) (20 ng per insect) were injected into late stage larvae and 0-1 d-old pupae, respectively. Proteins were extracted from pronotum and elytra dissected from five dsRNA-treated insects collected at the 0 d-old adult stage (A0) and analyzed by 15% SDS-PAGE, followed by Coomassie staining (left panel) or western blotting (right panel). Arrows in left panel indicate TcCP30-like proteins (note that they are absent in the ds*CP30* lanes). dsRNA for *TcVer* (ds*Ver*) (20 or 200 ng per insect) was injected as a negative control.

**Figure 5:**
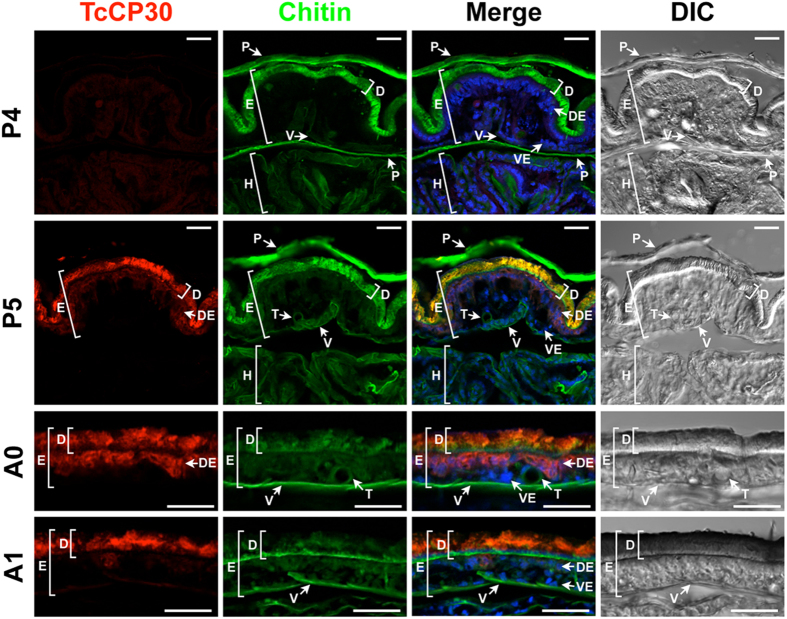
Localization of TcCP30 protein in elytral cuticle during development. Cryosections (~12 μm) of 4 and 5 d-old pupae and 0 to 1 d-old adults were incubated with the anti-TcCP30 antibody. Anti-TcCP30 antibody was detected by Alexa Fluor 546-conjugated anti-rabbit IgG antibody (red). FITC-conjugated chitin-binding probe (FITC-CBD) was used to stain cuticular chitin (green). Nuclei were stained with TO-PRO3 (blue). In merged images, yellow: TcCP30 and chitin, purple: TcCP30 and nuclei. P4, day 4 pupa; P5, day 5 pupa; A0, day 0 adult; A1, day 1 adult. E, elytron; D, dorsal elytral cuticle; V, ventral elytral cuticle; DE, dorsal elytral layer of epithelial cells; VE, ventral elytral layer of epithelial cells; H, hindwing; T, trachea; P, pupal cuticle. Scale bar = 20 μm.

**Figure 6:**
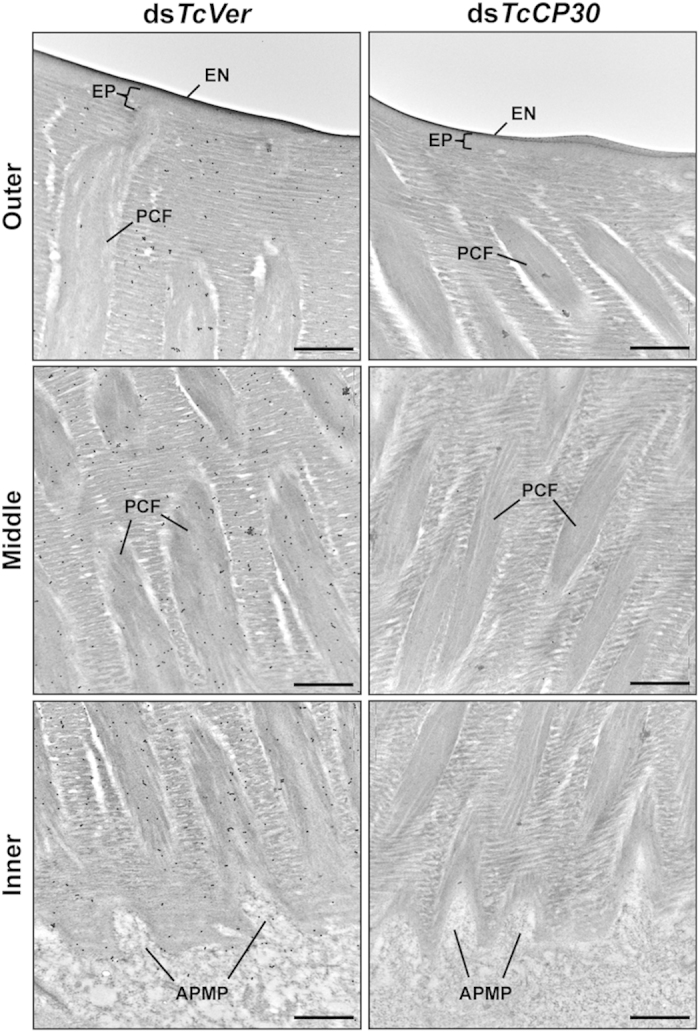
Localization of TcCP30 protein in dorsal elytral cuticle. Ultra-thin sections (~90 nm) of pharate adults (day 5 pupae) that had been injected with ds*TcCP30* or ds*TcVer* (200 ng per insects) at late larval stage were incubated with anti-TcCP30 antibody. The TcCP30 antibody was then detected using goat anti-rabbit IgG conjugated to 10 nm gold particles. TcCP30 protein is present in both horizontal chitin laminae and vertical PCFs of dorsal elytral cuticle of ds*TcVer*-treated control insects (left panels). Following RNAi for *TcCP30*, the abundance of gold particles is decreased in TcCP30-deficient insects (right panels) compared to ds*TcVer*-treated control insects. APMP, apical plasma membrane protrusions; EN, envelope; EP, epicuticle; PCF, pore canal fibers. Scale bar = 500 nm.

To investigate the function(s) of *TcCP30* in *T. castaneum*, we performed RNAi experiments to examine the effect of reduced expression of this cuticular protein gene. As a negative control, we utilized dsRNA for *T. castaneum Vermilion* (ds*TcVer*), a gene required for normal eye pigmentation^23^. Injection of dsRNA for *TcCP30* (ds*TcCP30*) substantially down-regulated expression of the *TcCP30* gene at both the mRNA (Fig. 2A) and protein levels (Figs. 3A, 4, 5, 6 and Supplementary Fig. 4). Injection of ds*TcCP30* (200 ng per insect) into late stage larvae had no effect on larval development, larval-larval or larval-pupal molting. The resulting pupae developed normally, and pigmentation of pupal cuticle including the setae, gin traps and urogomphi was normal (left panel in Fig. 2B). The adult cuticle pigmentation of head, mandibles, legs and pterostigma of the hindwings was initiated on schedule and visible through the old pupal cuticle (left panel in Fig. 2B). The subsequent pupal-adult molt, however, was adversely affected by *TcCP30* RNAi. Although apolysis and slippage were evident, some of the pharate adults (~70%) could not shed the pupal exuvium and died without undergoing eclosion (middle panel in Fig. 2B). In addition, those adults that did eclose exhibited wrinkled and separated elytra, resulting in improper folding of their hindwings (right panel in Fig. 2B).

### TcCP30 protein undergoes cross-linking

A polyclonal antibody against CP30 was generated using a synthesized peptide (E^160^-W^171^) (Fig. 1B) as the antigen, which corresponds to the C-terminal region of the TcCP30 protein. To improve antibody specificity, the TcCP30 antiserum was purified using an rTcCP30-immobilized affinity column. To verify the specificity of the purified TcCP30 antibody, western blot analysis of protein extracts from untanned elytra dissected from ds*TcVer*-treated control and ds*TcCP30*-treated day 5 pupae (pharate adults) was performed. The TcCP30 antibody detected only one major protein of the expected apparent mass (~30 kDa) (Figs. 3A and P5 in Fig. 3B) and several additional proteins of higher molecular mass (>37 kDa) in ds*TcVer*- but not in the ds*TcCP30*-treated insect extracts (Fig. 3A). The finding that TcCP30 proteins are substantially depleted after RNAi for this gene confirmed the specificity of the antibody (compare Coomassie staining pattern in ds*TcVer* lane with that of the ds*TcCP30* lane; in the left panel, position of TcCP30 is indicated by arrows).

To evaluate the TcCP30 cross-linking hypothesis, we extracted proteins from both the pronotum and elytra dissected from ds*TcVer*- and ds*TcCP30*-treated day 5 pupae. Tanning of the pronotum cuticle of the pharate adult occurs before eclosion, whereas tanning of the elytron occurs primarily after eclosion. Some pronotum CPs are already cross-linked at the time of the pupal-adult molt^21^. As shown in the right panel in Fig. 3A, the TcCP30 antibody detected several immunoreactive pronotum proteins of 30 kDa and higher molecular masses in the ds*TcVer* but not in the ds*TcCP30* protein extracts of the pronotum. Similarly, a 30 kDa protein and several larger immunoreactive proteins were detected in the protein extract from elytra dissected from wild-type day 0 adults (right panel in Fig. 3B). Those proteins, however, were undetectable in the day 1 adult protein extract (Fig. 3B). Since TcCP30 and several high molecular mass TcCP30-immunoreactive proteins were detected in untanned and partially tanned dorsal elytral cuticles, and were much less extractable in tanned adult cuticle from both the elytron and pronotum (Figs. 3 and 4B), we hypothesize that TcCP30 becomes rapidly cross-linked in rigid adult cuticles.

TcCPR4 (an RR-1 protein)^34^ and both TcCPR27 and TcCPR18 (RR-2 proteins)^21^,^22^ are abundant CPs found in rigid cuticles of the elytron, pronotum and ventral abdomen of adult *T. castaneum* (Figs. 3 and 4A). To evaluate whether these CPs are cross-linking partners of TcCP30, we analyzed the proteins extracted from elytra dissected from ds*TcCPR4-*, ds*TcCPR27*-, ds*TcCPR18-*, ds*TcCP30*- and ds*TcVer-*treated day 0 adults using SDS-PAGE. In addition to the 25 kDa TcCP30 protein, two TcCP30 immunoreactive proteins with apparent molecular masses of approximately 37 and 45 kDa were detected in the protein extracts of control ds*TcVer*-treated insects (arrows in Fig. 4A). Significantly, the 37 kDa immunoreactive protein was absent in TcCPR27-deficient insects, while the 45 kDa immunoreactive protein was absent in TcCPR18-deficient adults. The expected sizes for TcCP30-TcCPR27 (~37 kDa) and TcCP30-TcCPR18 (~43 kDa) conjugates are comparable to the molecular masses for these two major immunoreactive proteins. These results suggest that the 37 and 45 kDa immunoreactive proteins are conjugates of TcCP30 with TcCPR27 and TcCPR18, respectively. Furthermore, several proteins of higher molecular mass (>60 kDa) present in the ds*TcVer* and ds*TcCPR18* extracts were substantially reduced in the ds*TcCPR27* extract and absent in the ds*TcCP30* extract (bracket in Fig. 4A). No obvious difference was observed in the TcCP30-immunoreactive patterns between the proteins extracted from elytra dissected from ds*TcCPR4*- or ds*TcVer-*treated day 0 adults (Fig. 4A). This result suggests that TcCPR4 is not a cross-linking partner of TcCP30. All of these data indicate that TcCP30 becomes conjugated with both TcCPR27 and TcCPR18 (and possibly to other) proteins *in vivo*.

### Laccase 2 is involved in TcCP30 protein cross-linking

During cuticle tanning, cross-linking occurs as a result of oxidative and nucleophilic reactions between highly reactive quinonoids derived from *N*-acetyldopamine (NADA) or *N*-β-alanyldopamine (NBAD) and nucleophilic side chains of CPs^5^,^24^,^25^. We previously reported that catechol oxidation was catalyzed by a phenoloxidase, laccase 2 (TcLac2) in *T. castaneum*^3^. RNAi of *TcLac2* resulted in no larval, pupal or adult cuticle tanning, and the insects died prematurely. The critical role for Lac2 in cuticle tanning appears to be widely conserved among insect species^26^,^27^,^28^,^29^,^30^,^31^,^32^. To investigate the potential cross-linking of TcCP30, we produced recombinant TcCP30 (rTcCP30) in *E. coli*. When rTcCP30 was incubated in the presence of an insect laccase 2 and NBAD, a ladder of higher molecular mass proteins was produced, at sizes consistent with SDS stable, cross-linked multimers of TcCP30 (Supplementary Figs. 3B and 3C). The cross-linking reaction did not occur in the absence of NBAD, indicating that the product of NBAD oxidation by laccase 2 promotes the cross-linking of rTcCP30. Histidine residues are very likely to participate in quinone and/or quinone-methide cross-linking of catechols to CPs^24^. TcCP30 (21%), TcCPR27 (16%), and TcCPR18 (10%) all have a high histidine content, while TcCPR4 (3%) does not, suggesting that cross-linking of TcCP30 to TcCPR27 and TcCPR18 may occur via histidines.

To evaluate whether TcCP30 protein cross-linking in elytral cuticle requires TcLac2, we injected 20 ng ds*TcLac2* into day 0-1 pupae to induce a hypomorphic phenotype in adults^3^. The TcCP30 antibody detected a 30 kDa protein and several larger immunoreactive proteins in the protein extract of control ds*TcVer*-treated day 0 adult insects (brackets in Fig. 4B), whereas those proteins were greatly decreased in intensity in the protein extract of ds*TcLac2*-treated insects of the same age. This result indicates that TcLac2 greatly enhances the formation of the higher molecular weight proteins, which are likely to be formed by the cross-linking of TcCP30 to itself and/or other cuticular proteins including TcCPR27 and TcCPR18 by oxidized catechols. The persistence of small amounts of cross-linked proteins detectable in the ds*TcLac2*-treated insects may be due to incomplete elimination of the TcLac2 protein under the RNAi conditions that were employed to achieve a hypomorphic phenotype, rather than a lethal phenotype.

### Localization of TcCP30 protein

To determine the localization of TcCP30 protein in adult cuticle of *T. castaneum*, we conducted an immunohistochemical study of epidermal tissue. Cryosections (~12 μm) prepared from pharate adults (day 4 and 5 pupae) and young adults (day 0 and 1) were incubated with the TcCP30 antibody and FITC-CBD protein to label TcCP30 and chitin, respectively. In pharate adults (day 5 pupae), TcCP30 protein was found in cuticles of the dorsal elytron, ventral abdomen and thoracic body wall, which become highly sclerotized and pigmented as the adults matured (Fig. 5 and Supplementary Fig. 4) as well as in the epithelial cells underlying those cuticles such as elytron (see purple color in “Merge” panels in P5 and A0 in Fig. 5 and Supplementary Fig. 4B). Little or no TcCP30 protein was detected in cuticles associated with the ventral elytron, hindwing or dorsal abdomen, all of which are soft, membranous and relatively unpigmented (Fig. 5 and Supplementary Fig. 4). Tracheal cuticle was also devoid of TcCP30.

TcCP30 was undetectable in elytra of day 4 pupae, whereas it was readily detected in the dorsal elytral cuticle of day 5 pupae and also at later stages of development (Fig. 5). These results were consistent with the expression profiles of transcripts for the *TcCP30* gene (Supplementary Fig. 2). TcCP30 protein was co-localized with chitin in the procuticle of dorsal elytral cuticle (Fig. 5 and Supplementary Fig. 4B). The chitin signal in the dorsal elytral cuticle appears to be weaker after adult eclosion (panels A0 and A1 in Fig. 5), probably because of the cuticle becoming tanned, with the cross-linked CPs masking chitin and/or due to the pigments quenching FITC-CBD fluorescence.

To understand the function or importance of a particular CP in cuticle formation and in influencing its mechanical properties, it is critical to know the specific location of the protein within the cuticle. The technique of EM immunolocalization of CPs within the cuticle was introduced over a quarter century ago, and early data were summarized by Willis *et al* (2005)^20^. Recently, Vannini *et al*^33^ analyzed the location of AgamCPF3 and AgamCPLCG3/4 cuticular proteins by immunogold labeling TEM in adult cuticle of *Anopheles gambiae*. The AgamCPF3 protein is mainly localized in the exocuticle, whereas the two AgamCPLCG3/4 proteins are restricted to the endocuticle. The locations of these proteins appear to be correlated with the temporal expression of their genes; maximal levels of transcripts of the former were detected in the pharate adult, whereas transcripts for the latter two were maximal in the young adult after eclosion.

**Figure 7:**
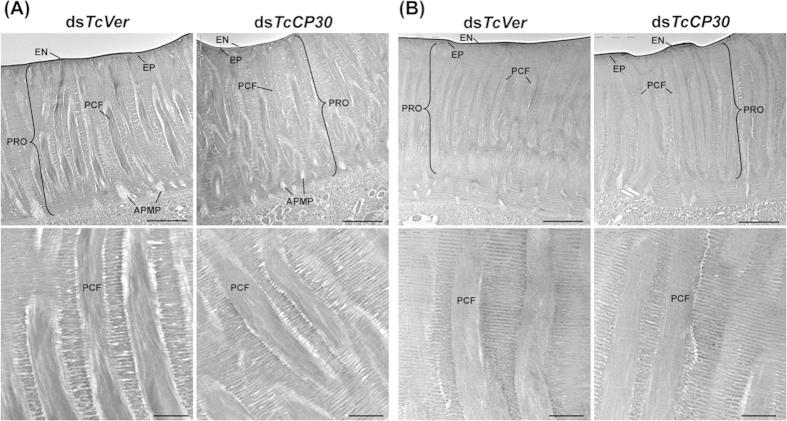
Ultrastructure of elytral cuticle of TcCP30-deficient adults. (**A**) Elytra from pharate adults (day 5 pupae) and (**B**) day 1 adults that had been injected with ds*TcCP30* or ds*TcVer* at late larval stage were collected for analysis of ultrastructure by TEM. Bottom panels show enlarged images of the horizontal laminae and vertical pore canals in the procuticle of the dorsal elytral cuticles from each of the dsRNA treated-insects. EN, envelope; EP, epicuticle; PRO, procuticle; PCF, pore canal fiber; APMP, apical plasma membrane protrusion. Scale bar in top panels = 2 μm and bottom panels = 500 nm.

We previously reported that three functionally distinct layers, the envelope, epicuticle and procuticle^1^,^2^, were evident in rigid cuticles such as those of the dorsal elytron, ventral abdomen, thoracic body wall and leg from the pharate adult (day 5 pupae) of *T. castaneum*^22^. In the procuticle, furthermore, there are not only numerous horizontally oriented chitinous laminae parallel to the apical plasma membrane of the epidermal cell but also numerous vertical columnar structures denoted as pore canals (PCs) containing vertically oriented chitinous fibers. These are denoted as pore canal fibers (PCFs) that extend directly from a region just above the apical plasma membrane protrusions (APMPs) of the underlying epidermal cells (Figs. 6 and 7A). Because there are far fewer horizontal laminae and no vertical PCFs in soft, flexible and less pigmented cuticles such as those of the dorsal abdomen, ventral elytron and hindwing^22^, these observations suggest that the dense arrangement of numerous compact laminae and PCFs contributes to the thickness and acquisition of greater mechanical strength of rigid cuticle relative to the thinner softer cuticles.

To determine a more precise localization of TcCP30 protein in dorsal elytral cuticle of *T. castaneum*, we performed immunogold labeling followed by TEM analysis. Previously, we reported that TcCPR27 protein was present throughout the procuticle in both the horizontal chitinous laminae and vertical PCFs, while TcCPR4 was mainly in the vertical PCFs^22^,^34^. Like TcCPR27, TcCP30 protein was localized in the horizontal laminae and in vertical PCFs, but not in the epicuticle or envelope layers (Fig. 6). The abundance of gold particles in the procuticle of dorsal elytral cuticle from TcCP30-deficient insects was substantially reduced relative to that of ds*TcVer*-treated control insects (Fig. 6).

Unlike ds*TcCPR4*-treated insects that exhibited abnormal and amorphous PCFs^34^, there was no obvious difference in the ultrastructure of the dorsal elytral cuticle between control ds*TcVer*- and ds*TcCP30*-treated pharate adults (day 5 pupae) or day 1 adults including the envelope, epicuticle, chitinous horizontal laminae and vertical pore canals as indicated by TEM analyses (Fig. 7). These results suggest that TcCP30 and its cross-linked conjugates are not involved in organizing chitin into horizontal laminae and/or vertical pore canal fibers. Additional experiments are needed to investigate the role of TcCP30 cross-linking on the mechanical strength of rigid cuticle of adult *T. castaneum*.

Of the four major extractable cuticular proteins in rigid cuticles of the red flour beetle, *T. castaneum*, two have now been identified as RR-2 proteins, one as RR-1 and the fourth as a low complexity protein without an R&R motif. All four have been shown by RNAi experiments to be of functional importance in morphogenesis and/or ultrastructure of the exoskeleton. Additionally, TcCPR27 and TcCPR18 apparently become cross-linked to TcCP30 largely through the action of laccase 2, which likely contributes to the loss of extractability of these proteins. Many other CPs are present in rigid and flexible beetle cuticles, and their importance in cuticle morphogenesis awaits further studies.

## Methods

### Insect culture

The GA-1 strain of *T.* castaneum was used in this study. Beetles were reared at 30 °C and 50% relative humidity under standard conditions^35^.

### Protein extraction

Elytra and pronotum dissected from five pharate adults (day 5 pupae) were homogenized in 150 μl of phosphate-buffered saline (0.01 M PBS, pH7.4) containing 5% SDS, 4 M urea, 10% glycerol and the Halt Protease Inhibitor Cocktail (Thermo scientific). The homogenates were centrifuged at 13,000 x g for 3 min and the supernatants were analyzed by SDS-polyacrylamide gel electrophoresis (SDS-PAGE) using 15% acrylamide gels.

### Cloning a full-length *TcCP30* cDNA

To clone a cDNA of *TcCP30*, template cDNA was prepared from total RNA isolated from pharate adults (day 5 pupae) by using the RNeasy Mini Kit (Qiagen). One microgram of total RNA was used to synthesize the first-strand cDNA using an oligo-(dT) primer (Invitrogen). A partial cDNA containing the predicted coding sequence for *TcCP30* (516 bp) was amplified by PCR using the gene specific primers 5’-GTC AAA CGT CTT GTA CCA AC-3’ and 5’-TCC TTC TTG TGG CGA TTT AC-3’. To obtain a full-length *TcCP30* cDNA, 5’- and 3’-RACE PCRs were performed using the SMARTer^TM^ RACE cDNA Amplification Kit (Clontech) according to the manufacturer’s instructions. The first PCR was carried out using the Universal Primer A mix 5’-CTA ATA CGA CTC ACT ATA GGG C-3’ along with the gene-specific primer 5’-TCC TTC TTG TGG CGA TTT AC-3’ for 5’-RACE and 5’ GTC AAA CGT CTT GTA CCA AC 3’ for 3’-RACE. For the second nested PCR, the Nested Universal Primer A Mix 5’-AAG CAG TGG TAT CAA CGC AGA GT-3’ together with the same gene specific primers used for the first PCRs were utilized to reach the 5’- and 3’-ends of the *TcCP30* cDNA. All PCR products were cloned into pGEM-T (Promega) and sequenced. The TcCP30 DNA sequence was deposited in GenBank (accession number KP330488).

### Developmental expression profiles of *TcCP30*

To analyze the expression profile of *TcCP30* during development, template cDNA was prepared from total RNA extracted from pools of 6–10 whole insects at various developmental stages from embryos to adults, and cDNA was synthesized as described above. Real-time PCR was performed as described previously^22^ using the primers 5’-GAA CGC GAA GAA GAA CGC CAT CAT-3’ and 5’-TCT TGT GGC GAT TTA CCA CTC CCT-3’. Transcripts of *T. castaneum* ribosomal protein S6 (*TcRpS6*) were amplified using the primer pair 5’-ACG CAA GTC AGT TAG AGG GTG CAT-3’ and 5’-TCC TGT TCG CCT TTA CGC ACG ATA-3’ to normalize for differences between the samples in the concentration of cDNA templates.

### RNA interference

dsRNA for *TcCP30* (ds*TcCP30*) was synthesized as described previously^36^,^37^ using the following pair of primers, 5’-(T7)-TAG GGA AGG CGG CGA AG-3’ and 5’-(T7)-TGC CAT CAT CAT CAT GA-3’, where T7 indicates the T7 RNA polymerase recognition sequence (TAA TAC GAC TCA CTA TAG GG). The size of ds*TcCP30* is 248 bp. ds*TcCP30* (200 ng per insect) was injected into late instar larvae and the treated insects were kept at 30 °C under standard conditions for visual monitoring. dsRNA for the *T. castaneum Vermilion* gene (ds*TcVer*) was synthesized and injected to serve as a negative control^23^,^38^. To determine the knockdown level of *TcCP30* transcripts, template cDNAs were synthesized from total RNA isolated from pools of three day 5 pupae.

### Immunoblotting

Polyclonal antiserum for TcCP30 was generated by immunization using a C-terminal synthesized peptide ^160^EEWGHGWGRREW^171^ (Fig. 1B) into rabbits (Young In Frontier Co., Ltd., Korea). To improve antibody specificity, the TcCP30 antiserum was purified using the purified rTcCP30-immobilized affinity column (AminoLink Plus Immobilization kit, Thermo Scientific) according to manufacturer’s instruction. To evaluate the specificity of the anti-TcCP30 antibody, western blot analysis was performed. Proteins were extracted from elytra and pronotum dissected from pools of five day 5 pupae or day 0 adults that had been injected as late stage larvae with ds*TcCP30* or ds*TcVer* (200 ng per insect). Protein extracts were analyzed by 15% SDS-PAGE followed by Coomassie staining or western blotting using the anti-TcCP30 antibody.

### Immunohistochemistry

To analyze protein localization of TcCP30, immunostaining was performed as described previously^37^. Cryosections (~12 μm) of day 5 pupae that had been injected with ds*TcCP30* or ds*TcVer* (200 ng per insect) at the late larval stage were prepared. The tissues were rinsed with PBST (0.01 M PBS, pH 7.4 containing 0.2% TWEEN 20), and then blocked with blocking buffer (PBS containing 2% bovine serum albumin) for 1 h at room temperature. Tissues were incubated with the anti-TcCP30 antibody (1:300 in blocking buffer) for 3 h at room temperature followed by washing with PBST three times for 5 min each. Then the tissues were incubated with Alexa Fluor 546-conjugated anti-rabbit IgG (Invitrogen) (1:300 in blocking buffer) as secondary antibody for 1 h at room temperature. After washing the tissues three times with PBST, the FITC-conjugated chitin binding domain probe (FITC-CBD, 1:300 in PBST)^36^ was applied and incubated at 4 °C overnight. The sections were washed with PBST three times for 5 min each at room temperature and then nuclei were stained with TO-PRO3 (1:1000 in PBST) (Invitrogen) for 1 h at room temperature. Tissues were observed using a confocal laser-scanning microscope (Olympus FV500) equipped with appropriate filters.

### Transmission electron microscopy

Pharate adults (day 5 pupae) and day 1 adults that had been injected with ds*TcCP30* or ds*TcVer* (200 ng per insect) at the late larval stages were collected and fixed in a mixture of 4% paraformaldehyde and 0.1% glutaraldehyde in 0.1 M sodium cacodylate buffer (pH 7.4) for 24 h at room temperature. TEM analysis and immunogold labeling were performed as described previously^22^. For immunogold labeling, ultrathin sectioned samples (~90 nm) were blocked with 0.01 M PBS (pH 7.4) containing 5% normal goat serum for 1 h, and then incubated with anti-TcCP30 antibodies (1:20) in 0.05 M PBS containing 3% nonfat milk and 0.02% TWEEN 20 overnight at 4 °C. The tissues were rinsed with 0.01 M PBS three times for 3 min each and 0.05 M TBS (Tris-buffered saline) (pH 7.6) three times for 3 min each at room temperature followed by incubation with the secondary antibody conjugated with 10 nm gold particles (1:20) (goat anti-rabbit IgG conjugated with 10 nm gold particles, Electron Microscopy Sciences) in 0.05 M TBS (pH 8.0) containing 0.05% fish gelatin (BB International, Cardiff, UK) for 2 h at room temperature. The tissues were washed with 0.05 M TBS five times for 5 min each, deionized water four times for 1 min each at room temperature, and then stained with 4% aqueous uranyl acetate for 10 min.

## Author Contributions

Y.A. supervised the project. S.M. and M.Y.N. performed experiments. N.D. expressed recombinant TcCP30 protein and carried out *in vitro* cross-linking experiment. M.Y.N., N.D., S.M., K.K, M.K. and Y.A. discussed results and wrote the manuscript. All authors reviewed the manuscript.

## Additional Information

**How to cite this article**: Mun, S. *et al.* Cuticular protein with a low complexity sequence becomes cross-linked during insect cuticle sclerotization and is required for the adult molt. *Sci. Rep.* **5**, 10484; doi: 10.1038/srep10484 (2015).

## Supplementary Material

Supplementary Information
