# Supplementary material for: Cuticular protein with a low complexity sequence becomes cross-linked during insect cuticle sclerotization and is required for the adult molt
Source: Sci Rep. 2015 May 21;5:10484. doi: 10.1038/srep10484 (PMC4440208; doi:10.1038/srep10484)

Supplementary information

Cuticular protein with a low complexity sequence becomes cross-linked during insect cuticle sclerotization and is required for the adult molt

Seulgi Mun[1], Mi Young Noh[1], Neal T. Dittmer[2], Subbaratnam Muthukrishnan[2], Karl J. Kramer[2], Michael R. Kanost[2] & Yasuyuki Arakane[1]

[1]Department of Applied Biology, Chonnam National University, Gwangju, Korea

[2]Department of Biochemistry and Molecular Biophysics, Kansas State University, Manhattan, Kansas, United States of America

Methods

Expression of recombinant TcCP30 (rTcCP30)

The coding region of *TcCP30*, minus the predicted signal peptide, was amplified by PCR using the forward primer 5'-CC ATG GCC AGC CCC CAT CAC GAG GA-3' with the reverse primer 5'-CTC TGA GGC GAT TTA CCA CTC CCT GCG T-3' and cloned into the *Nco*I and *Xho*I restriction sites of the expression plasmid pET-28a (Novagen). Protein expression was performed with BL21 Star (DE3) cells (Life Technologies) cultured at 37°C in LB medium containing kanamycin (50 μg/ml). Protein expression was induced by the addition of IPTG to a final concentration of 1 mM. The cells were lysed by sonication, and rTcCP30 was purified from the soluble fraction by a combination of nickel-affinity and anion exchange chromatographies (Ni-NTA agarose, Qiagen, and HiPrep Q FF, GE Healthcare, respectively); the high histidine content of the rTcCP30 protein mediated binding to the nickel column without the need for a histidine

tag. Elution from the anion exchange column was with a 50 mM to 1 M linear gradient of ammonium bicarbonate (pH 8); fractions containing rTcCP30 were pooled and lyophilized.

rTcCP30 *in vitro* cross-linking

The cross-linking time course (Supplementary Fig. S3B) was performed in a 25 µl volume reaction mixture containing 143 µg rTcCP30 (~0.3 mM), 0.4 µg recombinant *Anopheles gambiae* laccase 2[1], 20 mM sodium acetate buffer (pH 5) with or without 1.5 mM *N*-β-alanyldopamine (NBAD). Samples were incubated at room temperature, and 5 µl aliquots were taken at 1, 2, 4, 8 and 22 h. Cross-linking reactions using different amounts of recombinant proteins (Supplementary Fig. S3C) were performed in a 5 µl volume reaction mixture containing 3 to 30 µg of rTcCP30 and 0.04 to 0.4 µg of rAgLac2, 1.5 mM NBAD, and 20 mM sodium acetate buffer; samples were incubated at room temperature for 22 h. Reactions were terminated by the addition of 2 X SDS sample buffer and heating the samples at 95°C for 5 min. All samples were stored at -20°C until analysis by SDS-PAGE.

Figure Legends

Supplementary Fig. S1. Alignment of TcCP30 with C1761841. The amino acid sequence of TcCP30 (minus signal peptide) was aligned with the deduced amino acid sequence of transcript C1761841 from the giant northern termite, *Mastotermes darwiniensis* (Mdar) (GenBank accession number GAZE01460444.1). Sequences were aligned using ClustalW2[2]. Asterisks (*) identify identical amino acids; colons (:) identify conservative substitutions; periods (.) identify weak substitutions.

Supplementary Fig. S2. Expression profile of *TcCP30* gene during development. (A) Relative levels of transcripts of *TcCP30* were quantified at different development stages from embryo to adult. The cDNAs used for real-time PCR were prepared from total RNA extracted from whole beetles. The *TcCP30* gene was highly expressed at the pupal stage only. E, embryo; YL, young larvae; ML, mature larvae; PP, pharate pupae; P, pupae; A, mature adults (2-3 weeks-old adults). (B) To analyze more precisely the expression patterns of *TcCP30* gene, the developmental stages were expanded between the early pharate pupal to young adult stages. The transcripts for the *TcCP30* gene drastically increased in day 5 pupae and declined shortly after adult eclosion. PP0, day 0-1 pharate pupae; PP1, day 1-2 pharate pupae; P0, day 0 pupae; P1, day 1 pupae; P2, day 2 pupae; P3, day 3 pupae; P4, day 4 pupae; P5, day 5 pupae; A0, day 0 adults; and A1, day 1 adults. Expression levels for *TcCP30* are presented relative to the levels of expression at the earliest developmental stage analyzed (E or PP0). The transcript levels of the *T. castaneum* ribosomal protein 6 (*TcRpS6*) were measured to normalize for differences between samples in concentrations of cDNA templates. Data are shown as the mean value ± SE (n = 3). (C) To analyzed the transcript levels of *TcCP30* in the elytra and hindwings, total RNA was extracted from tissues of day 5 pupae (n = 10). Expression levels for *TcCP30* are presented relative to the levels of expression in elytra. An asterisk indicates a significant difference in transcript levels of *TcCP30* between elytra and hindwings (p = 3.2-E06, t-test). Data are shown as the mean value ± SE (n = 3).

Supplementary Fig. S3. *In vitro* cross-linking of recombinant rTcCP30. (A) Variable mobility of TcCP30 in SDS-PAGE. dsRNAs for *TcCP30* (ds*CP30*) or *TcVer* (ds*Ver*) (200 ng per insect) were injected into late stage larvae. Proteins extracted from elytra dissected from five

dsRNA-treated pharate adults (day 5 pupae) and analyzed by 15% SDS-acrylamide gels in Tris-glycine electrophoresis buffer (left panel) and NuPAGE 4-12% Bis-Tris gels with MES buffer (right panel). (B) rTcCP30 protein was incubated with recombinant *A. gambiae* laccase 2 (rAgLac2) either in the absence (-) or presence (+) of 1.5 mM NBAD. Aliquots were taken at various time points for analysis by SDS-PAGE in NuPAGE 4-12%. C, rTcCP30 only; L, laccase 2 (rAgLac2). (C) Cross-linking with various ratios of rTcCP30 to rAgLac2. The amount of rAgLac2 was kept constant while rTcCP30 was varied from 30 µg to 3 µg (lanes 3-6), or rTcCP30 was kept constant while rAgLac2 was varied from 0.2 µg to 0.04 µg (lanes 7-9, compare to lane 3). Cross-linking was observed in all reactions although the efficiency may be slightly reduced at lower rAgLac2 concentrations. Lanes 1 and 2 are controls containing rTcCP30 only or rAgLac2 only.

Supplementary Fig. S4. Localization of TcCP30 protein in cuticle of *T. castaneum* adult. (A) Cryosections of pharate adults (day 5 pupae) that had been injected with ds*TcCP30* or ds*TcVer* (200 ng per insect) at late larval stage were incubated with the anti-TcCP30 antibody, which was then detected by Alexa Fluor 546-conjugated anti-rabbit IgG antibody (red). FITC-conjugated chitin-binding probe (FITC-CBD) was used to stain cuticular chitin (green). Nuclei were stained with TO-PRO3 (blue). In merged images, yellow: TcCP30 and chitin, purple: TcCP30 and nuclei. P, pupal cuticle; E, elytron; H, hindwing; VA, ventral abdomen; DA, dorsal abdomen; L, leg. Scale bar = 200 µm. (B) Enlarged image showing partial cross-section. P, pupal cuticle; E, elytron; H, hindwing; T, thoracic body wall. D, dorsal elytral cuticle; V, ventral elytral cuticle; DE, dorsal elytral layer of epithelial cells; VE, ventral elytral layer of epithelial cells. Scale bar = 50 µm.

References

1 Gorman, M. J. *et al.* Kinetic properties of alternatively spliced isoforms of laccase-2 from *Tribolium castaneum* and *Anopheles gambiae*. *Insect Biochem. Mol. Biol.* **42**, 193-202 (2012).

2 Larkin, M. A. *et al.* Clustal W and Clustal X version 2.0. *Bioinformatics* **23**, 2947-2948 (2007).

Supplementary Fig. S1

```
TcCP30   SPHHEERRHEERRREEEKHHHHREGGEEGGRGREEEHHHHREEERKHHREEEERKHHHRE 60
Mdar     -------------DNDAKSKFSFTGGSLE----EEEEDHHEEEEVQGHREEEEVQGHHEE 43
                       :: * :.   **.      ***..**.*** : ****** : **.*

TcCP30   EEERKHREEEERHHHREEERKHHHREEEERHHHREREEERHHHHEGEEGGRGGGEEEGRG 120
Mdar     EEVRDHREEEDLQDHREEEEVQDHHEEDEGQDNHEEDSDAS------------------- 84
         ** *.*****: :.*****. :.*:**:* :.::*.:.:

TcCP30   GEEHWGRGGEEEGGRGGGEEEEWGHGWGRREW 152
Mdar     --------------------------------
```

Supplementary Fig. S2

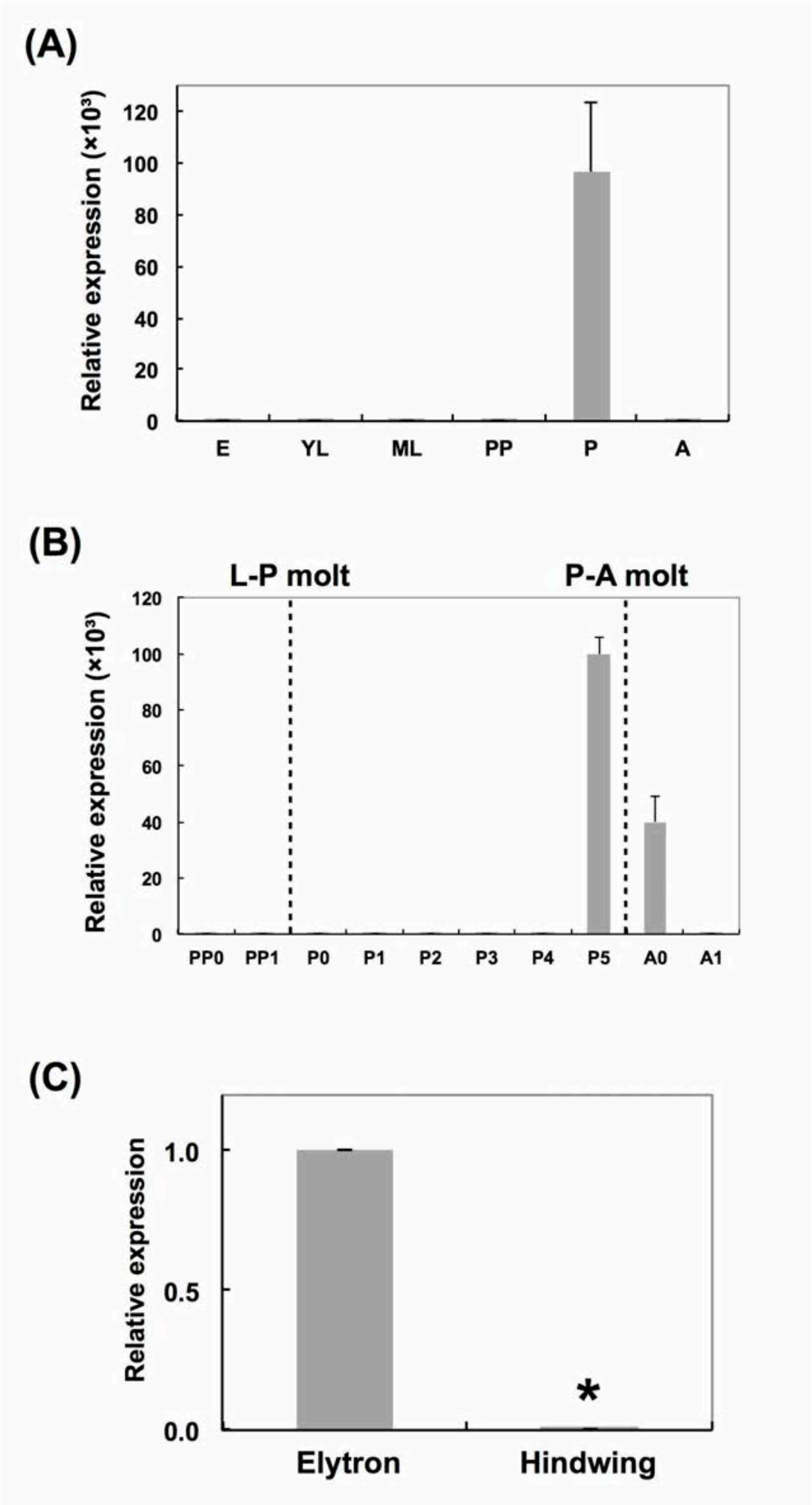

Supplementary Fig. S3

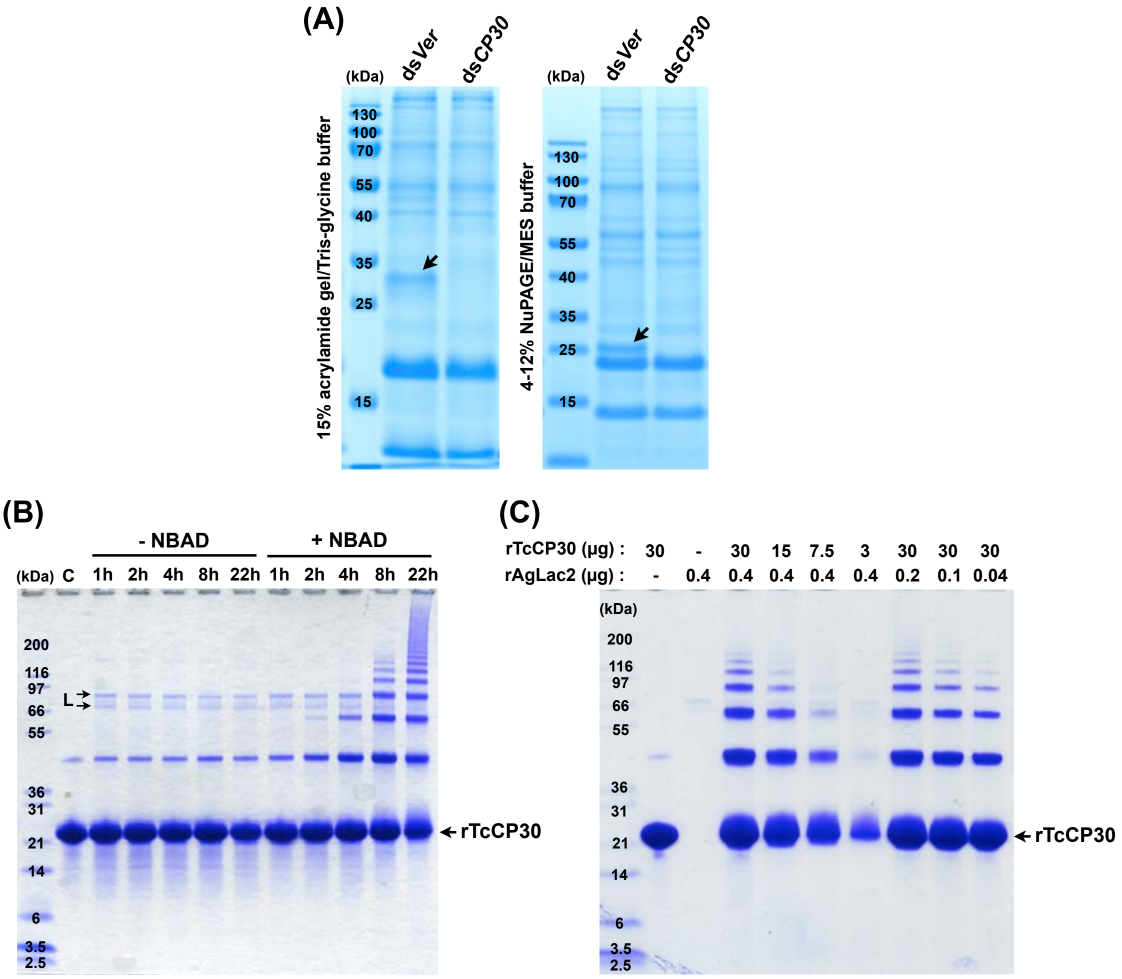

Supplementary Fig. S4

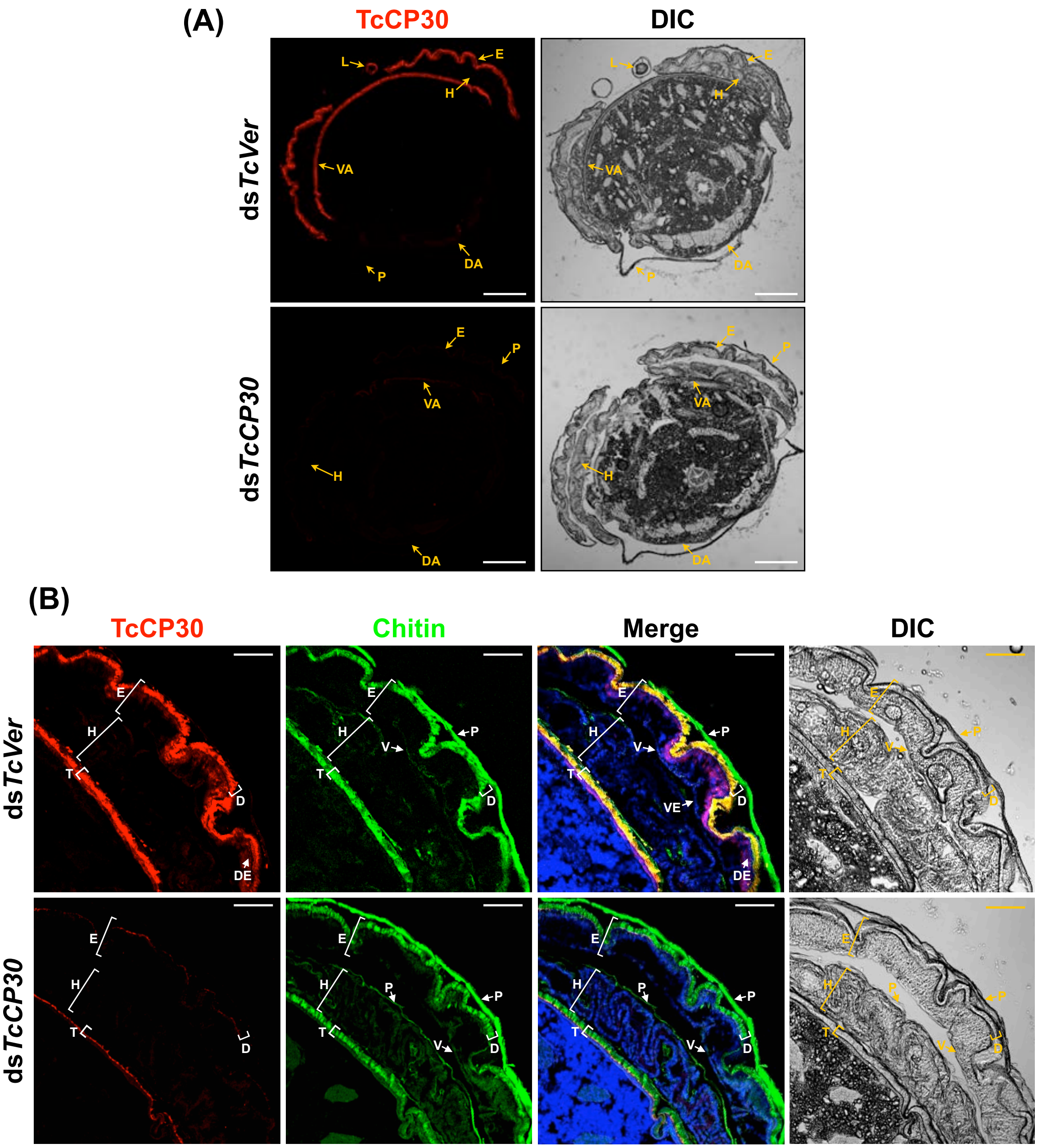

Supplement: Supplementary Information [file srep10484-s1.pdf]
